# Exploring Changes in Activity Patterns in Individuals with Chronic Pain

**DOI:** 10.3390/ijerph17103560

**Published:** 2020-05-19

**Authors:** Elena Rocío Serrano-Ibáñez, Rebecca Bendayan, Carmen Ramírez-Maestre, Alicia Eva López-Martínez, Gema Teresa Ruíz-Párraga, Madelon Peters, Rosa Esteve

**Affiliations:** 1 Facultad de Psicología, Universidad de Málaga, Instituto de Investigación Biomédica de Málaga, Andalucía Tech, 29071 Málaga, Spain; cramirez@uma.es (C.R.-M.); aelm@uma.es (A.E.L.-M.); gtruizparraga@uma.es (G.T.R.-P.); zarazaga@uma.es (R.E.); 2 Área de Psicología, Facultad de Ciencias de la Salud, Universidad Isabel I, 09003 Burgos, Spain; 3 Department Biostatistics and Health Informatics, Institute of Psychiatry, Psychology & Neuroscience (IoPPN), King’s College London, London WC2R 2LS, UK; rebecca.bendayan@kcl.ac.uk; 4 Department of Clinical Psychological Science, Faculty of Psychology and Neuroscience, Maastricht University, 6229 ER Maastricht, The Netherlands; madelon.peters@maastrichtuniversity.nl

**Keywords:** chronic pain, activity patterns, avoidance, persistence, pacing

## Abstract

This longitudinal study explored whether activity patterns change over time in a sample of 56 individuals with chronic musculoskeletal pain over a 15-day period. Once a day, the participants recorded their level of pain intensity and the degree to which they had engaged in several specific activity patterns. Linear mixed models with random coefficients were used to investigate the rate of change in the activity patterns. Age, sex, pain intensity, and pain duration were controlled. The results show that excessive persistence was the only self-reported activity pattern to show a linear change over the 15-day period. There was a decrease in excessive persistence, and this decrease was slower with higher levels of activity avoidance. However, no significant association was found between sex, age, pain intensity, and pain duration and excessive persistence at baseline or change over time. At baseline, a positive association was found between excessive persistence and pain avoidance, pain-related persistence, and pacing to reduce pain, and a negative association was found between excessive persistence and pacing to save energy for valued activities. This result suggests a profile characterized by alternate periods of high and low activity that, in this study, were unrelated to longitudinal changes in pain intensity.

## 1. Introduction

Activity patterns are consistent ways of behavior that shape how individuals organize their occupations [1]. Changes in these patterns typically accompany the experience of chronic pain [2] because individuals modify their activity to decrease their pain, maximize their functioning, or both [3]. These patterns play a relevant role in the development and maintenance of chronic pain [4]. An essential task in the rehabilitation process is to assess and, in most cases, change maladaptive activity patterns in order to improve patient functioning [5,6]. Thus, clinicians have to address the fundamental issue of the stability and consistency of activity patterns across different situations.

Traditionally, three activity patterns have been identified in individuals with chronic pain: persistence, avoidance, and pacing. Recently, more specific patterns have been distinguished [7,8]. Persistence has been differentiated into three types: (a) task-contingent persistence, in which individuals persist in finishing tasks despite pain; (b) excessive persistence, in which individuals do too much; and (c) pain-contingent persistence, in which the level of activity is determined by the pain at that moment. Avoidance has been differentiated into two patterns: (a) pain avoidance, which refers to avoidance behavior in the presence or anticipation of changes in pain; and (b) activity avoidance, which refers to the patient’s condition of being in pain rather than the fluctuating pain experience. Finally, three types of pacing have been differentiated according to the goal of the behavior: (a) to increase activity levels; (b) to conserve energy for valued activities; and (c) to reduce pain.

Cross-sectional studies have assumed that activity patterns are relatively stable, i.e., [8,9]. However, some results suggest that these patterns could vary depending on the situation and experience of the individual [10]. Therefore, longitudinal research is needed to explore this variability. Such research would be of assistance in the design and implementation of more effective interventions to regulate patient functioning and establish optimum activity patterns. Although some studies have classified participants according to their activity patterns and collected longitudinal data on their activity level using objective methods [11,12,13,14], longitudinal changes in self-reported activity patterns have not yet been systematically examined.

Individuals with chronic pain do not exclusively report one activity pattern, and the specific patterns seem to constitute larger configurations of activity [10]. Four subgroups have been identified [8,15]: (a) doers, who are characterized by high levels of task-contingent persistence, moderately high levels of excessive persistence and pain-related persistence, and low levels of the three types of pacing; (b) avoiders, who are characterized by high levels of pain avoidance, activity avoidance, and pacing to reduce pain, with low levels of the three types of persistence, pacing to do more things, and pacing to save energy for valued activities; (c) extreme cyclers, who have high levels of avoidance, moderately high levels of task-contingent persistence and excessive persistence, and low levels of the three types of pacing; and (d) medium cyclers, who have high levels of the three types of pacing and moderately high levels of pain avoidance, activity avoidance, task-contingent persistence, and excessive persistence. Extreme and medium cyclers were both characterized by high levels of pain-related persistence.

The aim of the present study was to explore whether self-reported activity patterns change over a 15-day period, during which a sample of individuals with chronic pain completed a diary. Any changes in activity patterns were analyzed for associations between them. We controlled the variables sex, age, pain intensity, and pain duration, because significant associations have been found between these variables and the frequency of use of the different patterns [16,17,18]. To the best of our knowledge, this study is the first one to explore whether these self-reported patterns change over time in individuals with chronic pain.

## 2. Materials and Methods

### 2.1. Procedure

Thirty-seven participants were recruited at two pain and physiotherapy units and 20 at two local fibromyalgia associations between March 2018 and December 2018. Individuals were considered eligible for inclusion if they met the following criteria: age between 18 and 65 years old; at the moment of participation they were experiencing pain and had had it for at least the last 6 months; they were not being treated for a malignancy, terminal illness, or psychiatric disorder. Two trained psychologists explained the aims of the study to the volunteer participants and how to fill in the diaries. The participants practiced by filling in an example diary. The participants were asked to fill in the diaries every day at night before going to bed. A reminder to fill in the diaries every day at the same hour was sent in their mobile phones.

This study was approved by the University of Málaga Ethics Committee and the Costa del Sol Health District. The participants were informed of the study aims, confidentiality was assured, and informed consent was obtained.

### 2.2. Participants

The sample comprised 56 individuals with chronic musculoskeletal pain with a mean age of 53.73 years (SD = 10.04). Of these participants, 51 were women, 75% were married or cohabiting, 28.6% reported an active working status; 39.3% had primary education or lower, 48.2% had secondary education, and 12.5% had higher education. These participants reported that their pain started on average 15.24 years before the study (SD = 13.07). Forty-eight percent of the sample reported generalized pain, 27% spinal pain, and 8% limb pain.

### 2.3. Variables and Instruments

#### 2.3.1. Demographic, Clinical, and Other Information

Information was collected on the demographic variables (sex, age, civil status, education level, work status) and clinical variables (pain duration and pain location).

#### 2.3.2. Pain Intensity

Participants were asked to rate their mildest, average, and worst pain during each day as well as their current pain on a scale ranging from 0 to 10, with a “0” indicating “no pain” and “10” indicating as “intense as you could imagine”. A composite pain intensity score was calculated for each day by calculating the average of the mildest, average, worst, and current pain [19].

#### 2.3.3. Activity Patterns

The eight activity patterns included in the Activity Patterns Scale [7] were assessed through 10 items: pain avoidance (2 items), activity avoidance (2 items), task-contingent persistence (1 item), excessive persistence (1 item), pain-contingent persistence (1 item), pacing to increase activity levels (1 item), pacing to conserve energy for valued activities (1 item), and pacing to reduce pain (1 item). The participants were asked to indicate to what extent the statement applied to the activity that they had performed each day on a 11-point scale ranging from 0 (not at all) to 10 (always).

### 2.4. Statistical Analysis

Preliminary descriptive and exploratory analyses were performed to examine associations at baseline using SPSS statistical software, version 22.0 for Windows. Linear mixed models were used to investigate the changes in activity patterns over time and potential interrelationships between activity patterns [20] using SAS Proc Mixed [21]. The main advantages of linear mixed models, via SAS Proc Mixed, are that they allow researchers to (a) include random factors (i.e., account for interindividual variability), (b) model the covariance structure of their data prior to testing the treatment effects, and (c) handle missing data [22]. An unstructured (UN) covariance structure was assumed and the intercept and slope were random. In order to ensure robust results with these sample sizes, we adjusted the degrees of freedom using the Kenward–Roger procedure, which has been found to be robust for samples of this size and distributional features (i.e., the procedure controls for potential inflated type I error rates) [23,24,25,26,27].

Linear unconditional and conditional models were fitted and adjusted for all the covariates considered: age, sex, pain duration, and pain intensity. The dichotomous covariates were effect-coded, and the baseline age and pain duration at baseline were mean centered. The model fit was estimated using the Bayesian Information Criterion (BIC) and −2LL. Intra-class coefficients (ICC) for the unconditional models were estimated as a measure of variation. ICCs of between 0.20 and 0.80 are suggestive of between and within-individual variations (i.e., differences between individuals and change over time). To facilitate the interpretation of the results, only significant parameter estimates are reported.

Missing data were imputed using Markov Chain Monte Carlo methods (MCMC); all analyses presented were conducted across 30 imputed data sets and combined using Rubin’s rules according to the percentages of missing data in each model [28,29,30]. Specifically, 53% of participants completed the diary for all 15 days, 14% completed it for 14 days, and 33% completed it for less than 14 days. There were no significant differences between the initial sample and the participants who completed 15 days in the demographic variables, clinical pain-related variables, and other variables included in our models. Sensitivity analyses were performed to compare complete and imputed cases and the results were similar [31].

## 3. Results

Table 1 shows the means and standard deviations for every activity pattern per day. No evidence was found of linear change in any of the activity patterns (Supplementary Materials, Table S1), except for the pattern of excessive persistence (Table 2). The ICCs are suggestive of a small variation over time in all the patterns; however, the model estimates show no evidence of a linear trend. Non-linear patterns could not be investigated given the moderate sample size. The excessive persistence pattern showed a slight linear decrease over time of 0.11 points per day. At the intercept level, a positive association was found between excessive persistence and pain avoidance, pain-related persistence, and pacing to reduce pain; a negative association was found between excessive persistence and pacing to save energy for valued activities. That is, individuals with higher scores in pain avoidance, pain-related persistence, and pacing to reduce pain also had higher scores in excessive persistence at baseline (first day of measurement); individuals with higher scores in pacing to save energy for valued activities had lower scores in excessive persistence. At the slope level, an association was found between excessive persistence and activity avoidance; that is, individuals with higher scores in activity avoidance showed a slower decrease in excessive persistence over time. The ICC for the excessive persistence pattern increased by 0.10 when conditional models were explored, suggesting that these patterns explain a proportion of the change in excessive persistence. Similar results were obtained after comparing the complete and imputed covariate data using a sensitivity analysis. The results showed that there was no significant association between age, sex, pain duration, and pain intensity at baseline and excessive persistence at the intercept and slope levels. The trajectories of pain intensity were investigated and no significant changes were found over time. Thus, pain intensity was only included at baseline in these models.

## 4. Discussion

The main aim of the present study was to explore the stability of or change in specific activity patterns in individuals with chronic musculoskeletal pain. Understanding how activity patterns function could be of relevance to rehabilitation professionals who work with chronic pain patients. The results of the present study showed that, with the exception of excessive persistence, there was no linear change in self-reported activity patterns over a 15-day period. Excessive persistence decreased over this period of time, and an association was found between its rate of change and the level of activity avoidance. However, no significant association was found between sex, age, pain intensity, and pain duration and excessive persistence at baseline or its decrease over time. The results show a positive association between excessive persistence at baseline and pain avoidance, pain-related persistence, and pacing to reduce pain, and a negative association between excessive persistence at baseline and pacing to save energy for valued activities.

Although it has generally been assumed that activity patterns are relatively stable, there has been no empirical confirmation of this core element of their definition, with the exception of some studies that have investigated the test-retest reliability of some activity pattern self-reports [32,33]. The results of the present study suggest that activity patterns can be conceptualized as general tendencies: that is, the way in which individuals habitually approach activity [34]. Thus, their assessment would facilitate the prediction of future behavior, which would be relevant to the objectives of rehabilitation [35,36].

The results show that excessive persistence was the only activity pattern that underwent a significant linear decrease over time. We suggest that this pattern is more labile than other patterns because the high level of activity that characterizes this pattern is unsustainable and tends to diminish over time. It should also be noted that the ICCs suggest that all the patterns may undergo small variations over time, which may follow nonlinear trends. Future studies should investigate these trends over time using larger sample sizes. Of note, the results showed an association between the decrease in excessive persistence and levels of activity avoidance, such that individuals with higher scores in activity avoidance showed a slower decrease in excessive persistence over time. As suggested in the early literature on this topic [37], we propose that both patterns are intertwined: periods of excessive persistence are followed by short periods of activity avoidance only when pain is extreme. This profile is characterized by peaks of activity that define individuals known as extreme cyclers [10,15]. We draw attention to a 7-day study that compared individuals with chronic pain and control individuals who continuously recorded their activity using an accelerometer. Although no differences were found between groups in the total amount of activity, differences were found in its distribution over the day. This result suggests a pattern of peaks in which individuals with chronic pain have higher activity levels in the morning and significantly lower activity levels in the evening than controls, who have a sustained activity rate over the day [14].

Previous studies have shown that periods of prolonged activity are followed by a significant increase in pain intensity (e.g., [17]). The results of a 5-day observational study showed that individuals who reported high levels of overactivity and high avoidance showed greater variations in pain and objective physical activity and also reported significantly higher levels of pain. However, the recurrence of prolonged periods of activity engagement followed by significant increases in pain was not observed in this group [11]. According to the results of the aforementioned studies, we could have expected a significant association between changes in excessive persistence and pain intensity; however, our study found no evidence of such an association. This result could be due to the fact that self-reported pain intensity appears to become stable once pain becomes chronic [38,39]. In addition, the present study found no evidence of linear changes in pain intensity. Future studies with larger samples could investigate the existence of a cross-lagged association between pain intensity and excessive persistence. In this sense, one study has shown that the relationship between the approach to activity engagement and lifting tolerance was affected by how long an individual had been experiencing pain; individuals who had been experiencing pain for 10 years or more reported a higher lifting tolerance than those who had been experiencing pain for 1 year [16]. The participants in the present study had experienced pain for an average of 15 years; thus, their pain intensity could be relatively stable and they may have developed greater tolerance to it, as indicated by them not perceiving or reporting significant fluctuations in self-reported pain intensity.

It has been suggested that extreme cyclers are context-dependent [18] and that, instead of pain intensity, the true disabling factor is fluctuations in their own activity level. The results of a 7-day diary study in a sample of individuals with chronic low-back pain showed no association between their mean activity level and their perceived level of disability; rather, a positive association was found between the intensity of activity fluctuations over time and the participants’ perceived level of disability [13]. Of note, the authors speculated that the disabling factor could be a lack of control regarding the performance of activities at any time. Previous studies have shown that after avoiders, extreme cyclers experienced the most disabling effects [15,16,18]. In fact, a recent 5-day dairy study showed that “avoiders” (low overactivity, high activity avoidance) and “mixed performers” (high overactivity, high activity avoidance) were quite similar in their participation in activity because both groups spent more time resting and less time in productive tasks than “overactives” [12].

The present study has a number of limitations. The only method used was self-reporting. This method could be influenced by memory bias, because the participants recorded their activity patterns once per day, and it could be also affected by social desirability. However, a 5-day observational study has shown that the information provided by self-report measures of overactivity and objective measures of physical activity were congruent [11]. Future research should not only use self-reports, but should also use objective measures of activity patterns [4]. Furthermore, activity patterns could be recorded several times per day instead of just once. No significant associations were found between age, sex, pain duration and pain intensity and a change in excessive persistence. This result could be due to the limited heterogeneity of the sample in relation to these covariates. Moreover, it was not possible to explore non-linear models due to the limited sample size; future studies with larger samples could investigate the presence of non-linear changes. Although the individuals of our sample have the typical profile of patients with longstanding chronic pain [40,41], their characteristics (i.e., mostly women and not currently working) could limit the generalizability of our results. In addition, our findings relate to patients with longstanding chronic pain who were followed over a relatively short follow-up period of 15 days; thus, future longitudinal research should include individuals with chronic pain of shorter duration and be conducted over longer follow-up periods.

## 5. Conclusions

Some studies on the rehabilitation process in patients with chronic pain have suggested that it is insufficient to eliminate dysfunctional patterns and implement functional ones in order to achieve effective adaptation; rather, it is crucial to change the relationship between activity patterns [2]. A recent study showed that a 12-week intervention changed the interrelationships between activity patterns [2]. The results showed that patients whose avoidance decreased while activity pacing remained unchanged or increased reported the highest level of functioning post-treatment, suggesting that interventions aimed at weakening the association between avoidance and activity pacing may enhance the functioning of individuals with ongoing pain. Specifically, the authors suggested that rehabilitation interventions that modify the function served by pacing behaviors—from avoiding pain to increasing productivity—may be particularly effective in weakening this association. Although these authors did not encounter the extreme cyclers profile in their study [2], the same suggestion could be applied to the relationship between the activity avoidance pattern and excessive persistence because, as the present study showed, changes in both patterns are interrelated. As mentioned, it has been speculated that the peaks in the activity characteristic of extreme cyclers could be context-dependent; that is, they could be determined by changing circumstances instead of by their own valued goals. In this sense, it has been suggested that the flexibility model—which is the model underlying acceptance and commitment therapy—could be a useful framework by which to understand and change activity patterns in chronic pain patients [42]. The flexibility model focusses on committed action, which is a concept that alludes to flexible behavior patterns guided by values and goals.

## Figures and Tables

**Table 1:** Patterns of activity per day. Means and standard deviations.

	Day 1 (Baseline)	Day 2	Day 3	Day 4	Day 5	Day 6	Day 7	Day 8	Day 9	Day 10	Day 11	Day 12	Day 13	Day 14	Day 15
Pain Avoidance-1	4.87 (2.53)	4.73 (2.79)	4.50 (2.88)	4.46 (2.70)	4.62 (3.18)	4.38 (2,69)	4.61 (2,35)	3.88 (2,43)	4.14 (2.97)	4.29 (2.49)	4.30 (2.51)	4.33 (2.52)	4.03 (2.31)	3.86 (2.25)	4.10 (2.42)
Activity Avoidance-1	4.43 (2.85)	5.06 (2.64)	4.77 (2.93)	4.44 (2.83)	4.13 (2.67)	3.77 (2.86)	4.77 (2.29)	4.10 (2.36)	3.45 (2.76)	4.61 (2.87)	4.21 (2.72)	3.95 (2.61)	3.92 (2.42)	3.92 (2.55)	4.27 (2.77)
Task-contingent Persistence	4.31 (3.14)	3.71 (2.64)	3.75 (2.94)	3.82 (2.71)	3.87 (2.63)	4.15 (3.01)	3.60 (2.53)	3.85 (2.91)	3.90 (3.05)	3.79 (2.76)	3.85 (2.67)	3.59 (2.58)	4.21 (2.47)	3.22 (2.86)	3.97 (2.87)
Excessive Persistence	4.72 (3.17)	4.65 (2.94)	4.29 (3.01)	4.37 (2.98)	4.07 (2.82)	4.20 (3.12)	4.09 (2.67)	3.76 (2.68)	4.38 (3.30)	4.57 (3.17)	4.08 (2.88)	4.15 (2.72)	3.84 (2.37)	3.81 (2.44)	4.40 (2.58)
Pain-related Persistence	4.21 (2.45)	3.60 (2.67)	4.01 (2.40)	2.96 (1.76)	3.01 (2.16)	3.31 (2.34)	3.48 (2.41)	4.01 (2.37)	3.16 (2.52)	3.64 (3.07)	3.59 (2.24)	2.96 (1.94)	3.64 (2.07)	3.92 (2.32)	4.21 (2.09)
Activity Avoidance-2	4.16 (3.11)	4.36 (2.83)	4.02 (2.89)	4.23 (3.04)	3.80 (3.13)	3.55 (2.91)	4.26 (2.54)	3.62 (2.40)	3.97 (3.09)	4.33 (3.03)	4.06 (2.95)	3.63 (2,72)	3.84 (2.65)	3.85 (2.46)	3.85 (2.56)
Pacing to do more things	5.26 (2.78)	5.18 (2.84)	4.58 (2.59)	4.29 (2.77)	4.62 (2.86)	4.25 (2.96)	4.84 (2.45)	4.39 (2.61)	4.45 (3.24)	4.31 (2.74)	4.68 (2.67)	4.45 (2.58)	4.39 (2.29)	4.36 (2.68)	4.57 (2.50)
Pacing to save energy for valued activities	4.76 (3.10)	5.27 (2.66)	4.38 (2.73)	4.39 (2.82)	4.35 (3.15)	4.43 (2.93)	4.63 (2.27)	4.32 (2.43)	4.57 (3.02)	4.48 (2.73)	4.39 (2.61)	4.33 (2.65)	4.82 (2.47)	4.47 (2,77)	4.73 (2.80)
Pacing to Reduce Pain	4.88 (3.06)	5.15 (2.68)	4.31 (2.89)	4.33 (2.87)	4.34 (3.03)	4.31 (2.85)	4.65 (2.44)	4.61 (2.73)	4.74 (3.11)	4.52 (2.83)	4.68 (2.91)	4.67 (2.72)	5 (2.43)	4.94 (2.84)	5.20 (2.55)
Pain Avoidance-2	6.06 (3.29)	5.72 (3.02)	5.21 (3.15)	4.90 (3.24)	5.37 (2.96)	4.76 (3.25)	5.49 (2.53)	4.66 (2.97)	4.98 (3.02)	5.05 (3.16)	5.28 (3.10)	4.85 (2.65)	5.39 (2.22)	4.61 (2.61)	4.87 (3.01)
Pain intensity	5.53 (1.73)	5.91 (1.80)	5.57 (1.92)	5.73 (1.71)	5.96 (1.76)	5.53 (1.89)	5.66 (1.79)	5.41 (1.76)	5.43 (2.14)	5.70 (1.71)	5.06 (1.72)	5.59 (1.76)	5.19 (1.61)	5.52 (1.77)	5.33 (1.80)

**Table 2:** Significant parameter estimates and fit indexes for the linear mixed model.

Model		
	Estimate (SE)	95%CI
**Intercept**	1.62 (0.45) ***	0.72, 2.52
Pain Avoidance−1 (baseline)	0.12 (0.05) **	0.02, 0.23
Pain−related Persistence	0.50 (0.04) ***	0.40, 0.59
Pacing to save Energy for Valued Activities	−0.21 (0.07) ***	−0.36, −0.07
Pacing to Reduce Pain	0.23 (0.07) ***	0.09, 0.38
**Slope**	−0.11 (0.03) **	−0.18, −0.05
Activity Avoidance−1	0.02 (0.07) ***	
**Variance**		
Intercept	3.07 (1.07) **	
Slope	0.002 (0.05)	
Residual	3.27 (0.26) ***	
**Fit Statistics**		
−2LL	1694.5	
BIC	1710.5	

** *p* < 0.1; *** *p* < 0.01

## Supplementary Materials

The following are available online at https://www.mdpi.com/1660-4601/17/10/3560/s1, Table S1: estimates for all the activity patterns.
