# Supplementary material for: Exploring Changes in Activity Patterns in Individuals with Chronic Pain
Source: Int J Environ Res Public Health. 2020 May 19;17(10):3560. doi: 10.3390/ijerph17103560 (PMC7277738; doi:10.3390/ijerph17103560)
Supplement: Supplementary file 1 [file ijerph-17-03560-s001.pdf]

Supplemental Table 1. Estimates for all the activity patterns.

	**Intercept**		**Slope**		**ICC**	**BIC**
	Estimate (SE)	95% CI	Estimate (SE)	95% CI		
Pain Avoidance - 1	4.76 (0.27)***	4.20, 5.31	−0.04 (0.02)	−0.09, 0.01	0.32	2942.4
Activity Avoidance - 1	4.67 (0.30)***	4.06, 5.29	−0.05 (0.03)	−0.11, 0.01	0.24	3048.3
Task-contingent Persistence	4.04 (0.31)***	3.41, 4.66	−0.02 (0.03)	−0.09, 0.03	0.30	3006.3
Excessive Persistence	4.54 (0.29)***	3.94, 5.14	−0.04* (0.02)	−0.09, 0.002	0.37	2997
Pain-related Persistence	3.85 (0.22)***	3.40, 4.31	−0.01 (0.02)	−0.06, 0.02	0.36	1780.9
Activity Avoidance - 2	4.18 (0.34)***	3.48, 4.87	−0.02 (0.03)	−0.09, 0.04	0.22	2800.7
Pacing to do more things	4.93 (0.30)***	4.31, 5.54	−0.03 (0.02)	−0.08, 0.01	0.46	2823.1
Pacing to save energy for valued activities	4.70 (0.30)***	4.09, 5.32	−0.005(0.02)	−0.05, 0.04	0.47	2842
Pacing to Reduce Pain	4.53 (0.29)***	3.93, 5.13	0.03 (0.02)	−0.009, 0.07	0.47	2847.9
Pain Avoidance - 2	5.60 (0.33)***	4.93, 6.27	−0.04 (0.02)	−0.08, 0.04	0.38	3035

* *p* <.05; ** *p* <.01; *** *p* <.001

ICC: Intra-class coefficients; *BIC*: Bayesian Information Criterion; *SE*: Standard Error; *CI*: Confidence Interval.
